# Association of Vitamin D Receptor Gene Polymorphisms with Cardiometabolic Phenotypes in Hispanics: A Life Course Approach

**DOI:** 10.3390/nu15092118

**Published:** 2023-04-28

**Authors:** Carrie S. Standage-Beier, Luis A. Garcia, Eleanna De Filippis, Gabriel Q. Shaibi, Lawrence J. Mandarino, Dawn K. Coletta

**Affiliations:** 1 School of Nutritional Sciences and Wellness, University of Arizona, Tucson, AZ 85721, USA; cstandagebeier@arizona.edu; 2 Center for Disparities in Diabetes, Obesity, and Metabolism, University of Arizona, Tucson, AZ 85724, USA; lagarcia1@arizona.edu (L.A.G.); mandarino@arizona.edu (L.J.M.); 3 Division of Endocrinology, Metabolism and Diabetes, Mayo Clinic Arizona, Scottsdale, AZ 85259, USA; defilippis.elena@mayo.edu; 4 Center for Health Promotion and Disease Prevention, Arizona State University, Phoenix, AZ 85004, USA; gabriel.shaibi@asu.edu; 5 Department of Medicine, Division of Endocrinology, University of Arizona, Tucson, AZ 85724, USA; 6 Department of Physiology, University of Arizona, Tucson, AZ 85724, USA

**Keywords:** vitamin D receptor, polymorphisms, SNPs, Latino, Hispanic, cardiometabolic traits

## Abstract

The vitamin D receptor (VDR) is vital for maintaining calcium and phosphate balance and regulating bone metabolism. Recent research has suggested that VDR also plays an essential role in metabolic diseases. Previous studies on non-Hispanic whites have shown that VDR single nucleotide polymorphisms (SNP) are associated with cardiometabolic phenotypes. However, the association between VDR SNPs and cardiometabolic traits in Hispanics remains unclear. This study investigated the association between VDR SNPs and cardiometabolic phenotypic data in self-reported Hispanics (n = 1610) from the Arizona Insulin Resistance registry and Sangre Por Salud Biobank. The study population was predominantly female (66.4%) with a mean age of 40 ± 14 years (n = 121 <18 years) and an average body mass index (BMI) of 29.8 ± 6.3 kg/m^2^. We performed a genotyping association analysis of VDR SNPs (Taq1-rs731236, Fok1-rs2228570 and Apa1-rs7975232) with cardiometabolic traits using linear regression models. The results showed that Taq1 and Apa1 were strongly associated with systolic blood pressure (SBP) in children (<18 years), while Fok1 was associated with measures of adiposity, including fat mass, waist circumference, and BMI. In age-stratified adult (≥18 years) models, Taq1 was strongly associated with hemoglobin A1c, while Apa1 was associated with BMI and fasting glucose. Fok1 had no significant associations in the adult models. In conclusion, the VDR SNPs were associated with several cardiometabolic phenotypes in this Hispanic sample, but the type and strength of the associations varied by age group.

## 1. Introduction

The vitamin D receptor (VDR) is a steroid hormone receptor superfamily member and ligand-activated transcriptional regulatory protein. With over 23,000 VDR binding sites found on the human genome thus far, the VDR is present in several body tissues [1,2]. Encoded by the VDR gene located on chromosome 12q and under the presence of 1,25-dihydroxy vitamin D [1,25(OH)_2_D_3_] it activates VDR, allowing for selective control of target cells’ expression [3,4]. VDR’s expression in intestinal, kidney, and bone cells makes it pivotal for calcium and phosphate homeostasis and bone metabolism [5]. Thus, vitamin D, a fat-soluble essential nutrient, and the VDR play crucial roles in several homeostatic processes in higher vertebrates such as humans.

Vitamin D’s metabolism is a complex multi-organ/tissue physiological process forming classical and non-classical pathways [6]. The classical pathway is involved in calcium homeostasis and targets the kidneys, gastrointestinal tract (GI), and bone. This pathway begins with vitamin D metabolized to either the conversion of pro-vitamin D3 (7-dehydro-cholesterol) with ultraviolet-B (UVB) radiation exposure to form vitamin D3 (cholecalciferol) in the skin or from dietary intake of vitamin D2 (ergocalciferol) and vitamin D3. Next, the vitamin D2 and D3 products undergo 25-hydroxylation in the liver, which forms calcifediol [25(OH)D], the primary circulating form of vitamin D. Calcifediol then undergoes 1α-hydroxylation in the kidneys, becoming vitamin D’s hormonally active form [1,25(OH)_2_D_3_]. Synthesis of [1,25(OH)_2_D_3_], in turn, activates the VDR, forming its non-classical functions and allowing for selective control of target tissues. These tissues include the skin, liver, kidney, heart, lungs, bladder, skeletal/smooth muscle, adipose, cartilage, and bones, thus regulating hormone secretion, immune function, and cellular proliferation/differentiation [7]. 

Fluctuations in vitamin D metabolism (including VDR expression) occur across the life course of an individual. These stages span from reproductive phases, including pregnancy and fetal health outcomes, to children’s growth and development, through adolescence, into naturally aging (elderly), and through disease [8,9,10,11]. Deficiencies in vitamin D can result in rickets in young children, osteomalacia in adults, and osteoporosis in the elderly [12]. More recently, vitamin D and the VDR have been connected to cardiometabolic-related disease pathogenesis such as cardiovascular disease (CVD), type 2 diabetes mellites (T2DM), and certain cancers [13,14]. Risk of developing these diseases has been connected to the development of cardiometabolic (metabolic) syndrome, a cluster of interrelated abnormal metabolic phenotypes, hereafter described as cardiometabolic phenotypes [15,16]. These phenotypes include dyslipidemia, elevated fasting blood glucose, high-density lipoproteins (HDL) cholesterol, hypertension, and abdominal obesity [17]. Conversely, studies have examined multiple vitamin D metabolism genes, including VDR single nucleotide polymorphisms (SNP), and their associations with vitamin D levels [14]. Obesity, a known risk factor for cardiometabolic diseases, has been found to be inversely associated with vitamin D levels across different life stages, ethnicities, and sex [18]. Further, associations have been found between deficiency of vitamin D’s major circulating metabolic [25(OH)_2_D_3_] and disease states, including CVD and T2DM [8]. 

Recent literature has linked VDR Taq1-rs731236, Fok1-rs2228570, and Apa1-rs7975232 VDR SNPs to cardiometabolic phenotypes [19,20,21]. This finding suggests that VDR gene polymorphisms have potential importance for cardiometabolic diseases such as CVD and T2DM. Studying VDR SNPs may help in understanding the genetic contributions to cardiometabolic disease pathophysiology. Our study focuses on Hispanics residing in the Southwestern United States (U.S.). Obesity and related diseases disproportionately impact children and adults from racial and ethnic minority subgroups, including Hispanics [22,23]. Thus, further examination of VDR SNP associations with cardiometabolic phenotypes in Hispanics is warranted to help better understand obesity-related health disparities in this key segment of the population. 

The importance of these VDR gene polymorphisms with cardiometabolic diseases remains controversial as associations have varied by type and strength between different ethnic and age groups [24]. Thus, the primary objective of this study was to elucidate further associations between the VDR (Taq1-rs731236, Fok1-rs2228570, and Apa1-rs7975232) SNPs and cardiometabolic phenotypes in a Hispanic sample population. Our secondary objective was to assess changes in the type and strength of associations between child and adult cohorts. We hypothesize that these VDR gene polymorphisms associate with cardiometabolic phenotypes; however, the associations will differ by age group.

## 2. Materials and Methods

### 2.1. Participants and Cohorts

Participants took part in either of the two cohorts. The first cohort, known as the Arizona Insulin Resistance (AIR) registry, consisted of Latino volunteers (n = 667) recruited through the community [25]. Most of the 667 volunteers were adults 18 years or older (79.6%); however, children and adolescents (20.4%) participated in the study. Participants received a history and physical examination, laboratory determinations, and a 75 g two-hour oral glucose tolerance test. Of these 667 participants, 94% consented to bank their DNA/serum and plasma for future studies without additional recontact. The second cohort consisted of Latino adults 18 years and older (n = 997) in the Sangre Por Salud (SPS) biobank [26]. SPS participants received laboratory determinations, a two-hour oral glucose tolerance test, and banked plasma/serum and DNA with consent to use deidentified data and biospecimens for future studies [26]. 

The IRB at Arizona State University approved the AIR registry study, and the IRB at Mayo Clinic approved the SPS biobank study. Written consent was obtained on all AIR registry and SPS biobank participants. Minors recruited into the AIR registry provided written assent with parents/guardians providing written consent. Consent was also given for banking serum, DNA, and RNA data for future studies like the one described herein. The University of Arizona approved the present study under protocol #1703255156. The present study was considered exempt by the ethics committee at the University of Arizona since it utilized deidentified information from previously consented banked samples and no recontact was made with those participants.

### 2.2. Single Nucleotide Polymorphism (SNP) Genotyping

Genotyping of the vitamin D receptor (VDR) Taq1-rs731236, Fok1-rs2228570, and Apa1-rs7975232 SNPs was performed by the Assay-by-Design service (Applied Biosystems, Foster City, CA, USA), as previously described [27,28]. Briefly, in a 384-well plate, 2 μL of purified genomic DNA (2 ng/μL) were incubated with primers and probes with the SNP of interest (0.09 μL), 3.5 μL of TaqMan Universal Polymerase Chain Reaction Master Mix-No AmpErase UNG, and 1.14 μL of distilled water. Samples were polymerase-chain-reaction-amplified on the Applied Biosystems 9700HT Thermal Cycler under the following conditions: denatured for 10 min at 95 °C, denatured, annealed, and extended for 40 cycles of 15 s at 92 °C and 1 min at 60 °C. We scanned the 384-well microplates for fluorescence emission using a 7900HT sequence detector (Applied Biosystems) and determined the alleles using the allelic discrimination Sequence Detection System v2.3 software (Applied Biosystems). 

### 2.3. AIR Registry Phenotypes Used for Genetic Analysis

The phenotypes used for the AIR registry’s VDR association analysis included body mass index (BMI), fat mass percentage (determined by bioimpedance; fat mass %), waist circumference (WC), hip circumference (HC), total cholesterol (Chol), triglyceride (TG), high-density lipoprotein-cholesterol (HDL), low-density lipoprotein-cholesterol (LDL), very low-density lipoprotein-cholesterol (VLDL), systolic blood pressure (SBP), diastolic blood pressure (DBP), alanine aminotransferase (ALT), aspartate aminotransferase (AST), adiponectin, fasting plasma glucose (FPG), 2-h oral glucose tolerance test (2hOGTT), hemoglobin A1c (HbA1c), fasting plasma insulin (FPI), prediabetes status and diabetes status using American Diabetes Association criteria. Collection and measurement of these phenotypes have been previously reported [25,26,28].

### 2.4. SPS Biobank Phenotypes for Genetic Analysis

Phenotypes used for the SPS biobank’s VDR association analysis included BMI, waist circumference (WC), total cholesterol (Chol), triglyceride (TG), high-density lipoprotein (HDL), low-density lipoprotein (LDL), fasting plasma glucose (FPG), 2-h oral glucose tolerance test (2hOGTT), hemoglobin A1c (HbA1c), and fasting plasma insulin (FPI). 

### 2.5. Statistical Analysis

We used R software, version 4.0.5 (https://www.r-project.org/, accessed on 1 July 2022) for the statistical analysis. Mean  ±  SEM was used to express participant characteristic data. Allele frequencies for VDR SNPs Taq1-rs731236, Fok1-rs2228570, and Apa1-rs7975232 were calculated in R using the genetics package (https://CRAN.R-project.org/package=genetics, accessed on 1 July 2022). This same package was used to test Hardy-Weinberg Equilibrium (HWE), and a threshold of *p* > 0.05 was used. We used the linear model function in R to create a simple regression model for the genetic association phenotype analysis. Welch’s *t*-test was used to test means between adult cohorts. Age and sex were used as covariates in the VDR variants phenotype association analyses. Significance was defined as *p* ≤ 0.05.

## 3. Results

Table 1 shows study characteristics stratified by age group (child < 18 years and adults ≥ 18 years) and cohorts (AIR registry and SPS biobank). We studied only those participants that had DNAs available for genotyping the VDR SNPs, as well as the phenotypic data available for the downstream analyses. As such, we had 990 SPS adult participants and, in the AIR registry, 499 adult and 121 child participants. Overall, we had 1610 participants with 66.4% females, mean age of 40 ± 14 years+ and average BMI of 29.8 ± 6.3 kg/m^2^. The overall genotype frequencies for Taq1 (AA: 950, AG: 568, GG: 88), Fok1 (GG: 460, GA: 796, AA: 354), and Apa1 (CC: 535, CA: 771, AA: 292) met HWE with *p* values of (*p* = 0.84), (*p* = 0.81), and (*p* = 0.65), respectively. Finally, linkage disequilibrium (LD) between SNPs were Taq1/Fok1 (D: −0.12, D′: 0.128), Taq1/Apa1 (D: 0.13, D′: 1.00), and Fok1/Apa1 (D: −0.04, D′: 0.20).

SPS biobank dataset was missing the following variables: fat mass %, HC, VLDL, SBP, DBP, ALT, AST, and adiponectin. BMI variation between AIR registry children, adults, and SPS biobank adults were 24.2, 29.8, and 30.5 kg/m^2^, respectively. As expected, WC, Chol, TG, LDL, FPG, and 2hOGTT mean values were higher in the adult cohorts than in the child cohorts. HbA1c was similar between the AIR registry children and adults at 5.5% and 5.6%, while the SPS biobank had the highest mean value at 6.1%. Additional phenotypic data in AIR registry participants showed an increase from children to adults in fat mass %, HC, VLDL, SBP, DBP, ALT, and AST. Though serum adiponectin was higher in children with 8.7 ug/mL than in adults at 6.4 ug/mL. 

Between the SPS and AIR (adult) biobanks, SPS had a slightly older population with a fairly equal distribution for gender. T-tests for phenotypes between groups were BMI (*p* = 0.04), WC (*p* = 0.01), Chol (*p* = 0.00001), TG (*p* = 0.07), HDL (*p* = 0.00001), LDL (*p* = 0.46), HbA1c (*p* = 0.00001), FPG (*p* = 0.41), and 2hOGTT (*p* = 0.00001).

Table 2 shows the cardiometabolic phenotypes that associated with VDR SNP Taq1, Fok1, and Apa1 in AIR registry children. Genotype frequencies for Taq1, Fok1, and Apa1 were AA: 74; AG: 44; GG: 2; AA; 29; AG: 64; GG: 28; and CC: 37; CA: 68; AA: 16, respectively. Taq1 strongly associated with SBP in both unadjusted (*p* = 0.0008) and adjusted (*p* = 0.005) models. Also, Fok1 associated with BMI, fat mass %, HC, WC, HC, and ALT, separately. We showed significant associations for fat mass % and WC in both unadjusted (*p* = 0.005 and *p* = 0.002) and adjusted (*p* = 0.007 and *p* = 0.004) models. Further, Apa1 associated with measures of blood pressure, where strong significant associations were observed for SBP in unadjusted (*p* = 0.003) and adjusted (*p* = 0.01) models.

Table 3 shows the cardiometabolic phenotypes that associated with VDR SNP Taq1, Fok1, and Apa1 in combined AIR registry and SPS biobank adults. Genotype frequencies for Taq1, Fok1, and Apa1 were AA: 876; AG: 524; GG: 86; GG: 432; GA: 732; AA: 325; and CC: 498; CA: 703; AA: 276; respectively. Taq1 was strongly associated with HbA1c in unadjusted (*p* = 0.005) and adjusted (*p* = 0.005) models. Fok1 had no significant associations. Apa1 was associated with BMI, WC, and FPG. The strongest associations were observed for BMI and FPG in both unadjusted (*p* = 0.03 and *p* = 0.04) and adjusted (*p* = 0.04 and *p* = 0.03) models.

## 4. Discussion

Study results showed VDR gene polymorphisms (Taq1, Fok1, and Apa1) associated with several cardiometabolic phenotypes in this Hispanic sample, though the type and strength of associations varied between children (<18 years) and adult (≥18 years) age groups. In child models, Taq1 and Apa1 were strongly associated with cardiac phenotypes SBP and DBP. Fok1 was strongly associated with body weight phenotypes of BMI, fat mass %, and WC, as well as ALT, a measure of liver function while, in adults, deviations were seen in both individual and combined AIR and SPS models. In AIR (individual) adult models, Apa1 was strongly associated with the glycemic phenotype and fasting plasma glucose, but Taq1 and Fok1 had no strong associations (see Appendix A, Table A1). In SPS (individual) adult models, Taq1 was strongly associated with a glycemic phenotype, hemoglobin A1c. Apa1 was strongly associated with body weight phenotypes of BMI and WC; Fok1 had no strong associations (see Appendix A, Table A2). In combined (AIR and SPS) adult models, Taq1 was strongly associated with hemoglobin A1c. Apa1 was strongly associated with BMI and fasting plasma glucose; Fok1 had no associations. Overall, these VDR gene polymorphisms were strongly associated with cardiac and body weight phenotypes in children, and with body weight and glycemic phenotypes in adults within this sample. 

Gene polymorphisms (SNPs) can arise from the germline substitution of a single nucleotide base pair on a (gene-specific) DNA sequence during DNA replication. Alterations in these allele frequencies, at a locus, appear in ≥1% of a population and can be due to point mutations in either the coding or non-coding regions of a gene. The locations of these VDR SNPs have been described as follows. Taq1 is located on the 3′ noncoding sequence in exon 9 of the ligand binding domain [29]. Fok1 is located on the exon 2 coding sequence of the DNA-binding (zinc fingers) domain [29]. Finally, Apa1 is on the 3′, intron 8 part of the ligand binding domain [29]. These site-dependent alterations to the VDR gene can have upstream impacts including Fok1, which has been found to have a significant relationship with serum 25(OH) [30]. Fok1 shortens the VDR transcription start site by three codons which, in turn, can cause a lower transcriptional activation of the VDR [6]. The literature has additionally suggested this connection to the renin-angiotensin-aldosterone system (RAAS), a determinate in hypertension, a cardiometabolic phenotype [31].

Limited studies have examined VDR gene polymorphisms’ associations with cardiometabolic phenotypes in Hispanic, Latino, or Mexican children and/or adults. Available literature though has shown that these VDR gene polymorphisms had mixed associations with cardiometabolic phenotypes and across the life course, consistent with our study results [13,14]. Differences in the strength of associations between ethnic groups are supported by larger systematic reviews and or meta-analyses [19,20,21]. Association studies in adults were more diverse than those in children and showed that these gene polymorphisms varied in strength of associations to different obesity/cardiometabolic phenotypes [24,32,33,34,35,36,37].

In our child sample, we found variations in genetic risk and protective factors. Taq1s dominant genotype (AA homozygous) showed the highest mean SBP of 113.1 mmHg, while both the heterozygous genotype (AG heterozygous) and recessive genotype (GG homozygous) had lower mean SBP values of 106.8 and 95.3 mmHg, respectively. All genotype means were within normal ranges for blood pressure [38,39]; however, the recessive Taq1 genotype was protective. Interestingly, for Fok1, BMI, fat mass %, WC, and HC, all showed the lowest mean from the recessive genotype (GG homozygous) group, while the dominant genotype (AA homozygous) had the highest means for BMI, fat mass %, WC, HC, and ALT. Therefore, the recessive Fok1 genotype was protective with obesity phenotypes and for measures of liver function (i.e., ALT). A study of Han children in China found that Fok1’s “AA” genotype significantly increased their risk for metabolic syndrome [40]. The sample genotype frequencies from the Han et al. study were Taq1 (TT: 0.87, TC: 0.11, CC: 0.02), Fok1 (GG: 0.27, GA: 0.50, AA: 0.23), and Apa1 (CC: 0.48, CA: 0.39, AA: 0.13) [40]. Our study findings did not replicate the Han children study as we showed a reduction in risk for obesity in the “AA” carriers. Differences in findings may, in part, be due to reduced transcriptional activation altering VDRs’ normal function coupled with Chinese populations having an increased risk for metabolic syndrome with a lower BMI [6,40,41,42]. These findings suggest that Chinese and Hispanic populations’ genetic profiles vary in their influence on cardiometabolic disease pathogenesis. Differences were also seen again when Apa1 “AA” genotype was associated with higher percentiles of BMI. In this present study, we found that Apa1 was strongly associated with cardiac phenotypes, with the recessive genotype (AA homozygous) showing the lowest means for SBP and DBP. The dominant genotype (CC homozygous) had the highest values for SBP and DBP, indicating that the minor allele was protective in our Hispanic sample.

Variations in genetic risk and protective factors were also seen in combined adult models. Taq1’s recessive genotype (GG homozygous) showed the lowest mean HbA1c value of 5.7%, while the dominant genotype (AA homozygous) had the highest mean HbA1c value of 6.0%. In turn, this recessive Taq1 genotype proved to play a protective role in blood glucose management. In a mixed Mexican sample, Rivera-Leon et al. found that Taq1’s dominant genotype was associated with low osteocalcin (a calcium-dependent biomarker determining bone turnover) levels in only overweight/obese individuals [43]. The sample genotype frequencies from the Rivera-Leon et al. study were Taq1 (TT: 0.30, Tt: 0.50, tt: 0.20) and Apa1 (AA: 0.38, Aa: 0.51, aa: 0.11) [43]. For our study, none of Taq1’s (and Apa1 or Fok1) genotypes were associated with osteocalcin in unadjusted or fully adjusted AIR registry adult and child models (not available for SPS). 

Conversely, Apa1’s recessive genotype (AA homozygous) had the highest mean values for BMI and WC compared to the dominant genotype. However, the recessive genotype for Apa1 also associated significantly with lower FPG levels. This association means that Apa1’s recessive genotype played a protective role in obesity phenotypes, whereas the recessive genotype was protective in blood glucose management. Finally, for Fok1, we found no associations in either individual (AIR or SPS) or combined (AIR + SPS) models. Despite another sample of Mexican participants, Piña-Aguero et al. found that Fok1’s “TT” genotype was associated with reduced beta cell function [44]. The sample genotype frequency from the Piña-Aguero et al. study for Fok1 was CC: 0.35; CT: 0.45; and TT: 0.20 [44]. Other literature in non-Hispanic adult populations also showed Fok1 having limited to no association with several cardiometabolic phenotypes [32].

Hispanic populations residing in the Southwest U.S. have been found to be more vitamin D deficient, despite high sun exposure, than non-Hispanic whites [45]. Vitamin D deficiency is traditionally defined as having a serum calcifediol level of <20 ng/mL [46]. Analysis of serum calcifediol levels in the 2001–2018 National Nutrition and Health Examination Surveys (NHANES) data on Americans >1 year old found that 2.6% and 22.0% were moderately and severely vitamin D deficient, respectively [47]. Race/ethnicity was also a strong independent risk factor for vitamin D deficiency; non-Hispanic whites had the lowest risk compared to Mexican Americans, individuals of other Hispanic origins, or non-Hispanic Black individuals [47]. This finding is consistent with the previous literature looking at different racial/ethnic populations with darker skin phenotypes, including Hispanics and African Americans [48,49]. As the intensity of melanin pigmentation (darkening of the skin) increases, it can reduce the level of pro-vitamin D3 converted by UVB to vitamin D3 [6]. However, geographic location, time of year, clothing or sunscreen use, dietary intake, vitamin D supplementation, and genetic variations can all play a role in how much vitamin D is metabolized. Genetic variants, Taq1 and Fok1, have been found to potentially improve metabolic response to vitamin D supplementation versus the Apa1 gene polymorphisms [50]. Although our study did not measure vitamin D levels, we postulate that the VDR gene polymorphisms in Hispanic populations may be a factor influencing vitamin D metabolism, along with other environmental exposures. 

Strengths of the present study included having <18 year and ≥18 year age groups in the study sample. Having both age groups allowed for examining deviations between VDR gene polymorphisms in children versus adults, a factor which was limited in the available literature. Conversely, study limitations include not having available data on serum vitamin D levels or other VDR SNPs, including Bsm1, in this sample. These data would have allowed for a more robust analysis of VDR SNPs and Vitamin D’s potential associations with cardiometabolic disease risk. Additionally, not knowing pubertal stage in children, as major metabolic/physiological changes occur between childhood and adolescence, limited our analysis. Having a <18 year age group does not account for lifecycle changes, potentially skewing the data. Lacking sun exposure and diet data also limited determining covariant associations to these VDR gene polymorphisms. Finally, the sample size was a limitation as a larger sample would have allowed for enough statistical power to detect cardiometabolic phenotype associations more precisely in both child and adult age groups. 

To our knowledge, this is one of the first studies looking at VDR gene polymorphisms association with cardiometabolic phenotypes in a combined sample of Hispanic adults and children. Our study’s data support both the larger examination of VDR’s importance in cardiometabolic disease pathogenesis and more specifically in Hispanic adult and child populations. Despite the available literature’s inconsistency regarding why VDR gene polymorphisms differ between ethnic groups, this study’s data support VDR’s potential role in cardiometabolic disease etiopathogenesis in Hispanic populations. Interestingly, we also found strong associations with the ectodysplasin A receptor (EDAR) SNP, EDARV370, cardiometabolic traits, and breast density in the AIR and SPS biobanks [51]. Research by Hlusko et al. suggests that EDARV370 effects on mammary gland development may be advantageous for those living at high altitudes during the population migration into the Americas [52]. That work also highlights its potential role in breast development and the potential role of transfer of nutrients and vitamins, such as Vitamin D, during lactation to infants [52], suggesting the VDR’s may play a potential role in maternal/child development, warranting further targeted research.

## 5. Conclusions

Our study results highlight that these VDR gene polymorphisms appeared to be either genetic protective or risk factors for different cardiometabolic phenotypes across the life course in this Hispanic sample. Children had the most associations overall, notably with adiposity phenotypes, while associations with adults were limited and mixed. Overall, the VDR SNP associations suggest a potential role in cardiometabolic disease pathophysiology across the life course in Hispanics. More targeted research is warranted to determine how these gene polymorphisms influence cardiometabolic phenotypes in different age groups among Hispanics, a population still burdened by cardiometabolic diseases. 

## Figures and Tables

**Table 1:** Characteristics of AIR registry and SPS biobank participants by age group.

Phenotypes	AIR–Children (<18 Age, Years)	AIR–Adults (≥18 Age, Years)	SPS–Adults (≥18 Age, Years)
	n = 121	n = 499	n = 990
Gender (Female/Male)	61/60	322/177	686/304
Age, years	14.1 ± 0.2	36.4 ± 0.5	45.8 ± 0.5
Body Mass Index, kg/m^2^	24.2 ± 0.6	29.8 ± 0.3	30.5 ± 0.3
Fat Mass, %	15.5 ± 1.0	24.3 ± 0.5	-
Waist Circumference, cm	83.3 ± 1.6	98.6 ± 0.6	100.7 ± 0.7
Hip Circumference, cm	97.4 ± 1.4	108.6 ± 0.5	-
Cholesterol, mg/dL	143.8 ± 2.7	174.3 ± 1.6	186.3 ± 1.6
Triglycerides, mg/dL	92.1 ± 4.5	136.5 ± 3.5	144.8 ± 4.2
High-density lipoprotein, mg/dL	45.2 ± 0.9	44.0 ± 0.5	49.96 ± 0.7
Low-density lipoprotein, mg/dL	82.8 ± 2.2	106.9 ± 1.3	108.1 ± 1.4
Very low-density lipoprotein, mg/dL	15.5 ± 0.7	21.8 ± 0.5	-
Systolic blood pressure, mm Hg	110.6 ± 1.1	120.1 ± 0.7	-
Diastolic blood pressure, mm Hg	67.5 ± 0.8	76.4 ± 0.4	-
Alanine aminotransferase, IU/L	17.2 ± 0.8	26.4 ± 0.8	-
Aspartate aminotransferase, IU/L	22.2 ± 0.7	24.0 ± 0.5	-
Adiponectin, ug/ml	8.7 ± 0.3	6.4 ± 0.1	-
Hemoglobin A1c, %	5.5 ± 0.03	5.6 ± 0.02	6.1 ± 0.1
Fasting plasma glucose, mg/dL	91.3 ± 0.6	94.4 ± 0.5	95.2 ± 1.1
2hOGTT, mg/dL	119.3 ± 2.8	136.96 ± 2.3	122.2 ± 2.9

Values are mean ± SEM. The - indicates phenotype not in dataset.

**Table 2:** AIR registry children (<18 age, years), genotype class-specific values for phenotypes significantly associated with Taq1, Fok1, and Apa1.

**Taq1-rs731236**	**AA** 74 (0.62)	**AG** 44 (0.37)	**GG** 2 (0.02)	*p*-value *	*p*-value **
Systolic blood pressure, mmHg	113.1	106.8	95.3	0.0008	0.005
**Fok1-rs2228570**	**AA** 29 (0.24)	**AG** 64 (0.53)	**GG** 28 (0.23)	*p*-value *	*p*-value **
Body mass index, kg/m^2^	26.6	23.9	22.2	0.01	0.02
Fat mass, %	20.7	14.4	12.4	0.005	0.007
Waist circumference, cm	92.8	81.1	78.4	0.002	0.004
Hip circumference, cm	102.9	96.4	93.8	0.01	0.03
Alanine aminotransferase, IU/L	21.7	15.5	16.5	0.03	0.03
**Apa1-rs7975232**	**CC** 37 (0.31)	**CA** 68 (0.56)	**AA** 16 (0.13)	*p*-value *	*p*-value **
Systolic blood pressure, mmHg	114.2	110.3	103.9	0.003	0.01
Diastolic blood pressure, mmHg	69.7	67.0	64.6	0.03	0.05

The *p*-values were generated using linear regression models in R software; n = sample number, and () is the % of the sample. * Genotype was only included in the liner regression model. ** Genotype, age, and sex were included in the linear regression model.

**Table 3:** AIR registry + SPS biobank combined adult (≥18 age, years) genotype class specific values for phenotypes significantly associated with Taq1, Fok1, and Apa1.

**Taq1-rs731236**	**AA** 876 (0.59)	**AG** 524 (0.35)	**GG** 86 (0.06)	*p*-value *	*p*-value **
Hemoglobin A1c, %	6.0	5.9	5.7	0.005	0.004
**Fok1-rs2228570**	**AA** 432 (0.29)	**AG** 732 (0.49)	**GG** 325 (0.22)	*p*-value *	*p*-value **
	NS	NS	NS		
**Apa1-rs7975232**	**CC** 498 (0.34)	**CA** 703 (0.48)	**AA** 276 (0.19)	*p*-value *	*p*-value **
Body mass index, kg/m^2^	30.0	30.2	31.0	0.03	0.04
Waist circumference, cm	99.5	99.6	102.0	0.04	0.05
Fasting plasma glucose, mg/dL	96.7	94.0	94.2	0.04	0.03

The *p*-values were generated using linear regression models in R software. NS = not significant. n = sample number, and () is the % of the sample. * Genotype was only included in the liner regression model. ** Genotype, age, and sex were included in the linear regression model.

## Data Availability

All relevant data are within the paper.

## Appendix A

**Table A1:** AIR registry adult (≥18 age, years), genotype class-specific values for phenotypes strongly associated with Taq1, Fok1, and Apa1.

**Taq1-rs731236**	**AA** 290 (0.58)	**AG** 185 (0.37)	**GG** 23 (0.05)	***p*-value ***	***p*-value ****
	NS	NS	NS		
**Fok1-rs2228570**	**GG** 157 (0.31)	**GA** 225 (0.45)	**AA** 117 (0.23)	***p*-value ***	***p*-value ****
	NS	NS	NS		
**Apa1-rs7975232**	**CC** 154 (0.31)	**CA** 263 (0.53)	**AA** 76 (0.15)	***p*-value ***	***p*-value ****
Fasting plasma glucose, mg/dL	96.3	93.7	93.5	0.04	0.02

The *p*-values were generated using linear regression models in R software. NS = not significant. n = sample number, and () is the % of the sample. * Genotype was only included in the liner regression model. ** Genotype, age, and sex were included in the linear regression model.

Table A1 shows the cardiometabolic phenotypes that strongly associated with VDR SNP, Taq1, Fok1, and Apa1 in AIR registry adults. Genotype frequencies for Taq1, Fok1, and Apa1 were (AA: 290, AG: 185, GG:23), (GG: 157, GA: 225, AA: 117), and (CC: 154, CA: 263, AA: 76), respectively. Taq1 and Fok1 had no strong associations. Apa1 strongly associated with FPG in unadjusted (*p* = 0.04) and adjusted (*p* = 0.02) models.

**Table A2:** SPS biobank adult (≥18 age, years), genotype class specific values for phenotypes strongly associated with Taq1, Fok1, and Apa1.

**Taq1-rs731236**	**AA** 586 (0.59)	**AG** 339 (0.34)	**GG** 63 (0.06)	***p*-value ***	***p*-value ****
Hemoglobin A1c, %	6.2	6.0	5.8	0.01	0.007
**Fok1-rs2228570**	**GG** 275 (0.28)	**GA** 507 (0.51)	**AA** 208 (0.21)	***p*-value ***	***p*-value ****
	NS	NS	NS		
**Apa1-rs7975232**	**CC** 344 (0.35)	**CA** 440 (0.45)	**AA** 200 (0.20)	***p*-value ***	***p*-value ****
Body mass index, kg/m^2^	30.0	30.4	31.5	0.005	0.006
Waist circumference, cm	99.8	100.3	103.4	0.01	0.01

The *p*-values were generated using linear regression models in R software. NS = not significant. n = sample number, and () is the % of the sample. * Genotype was only included in the liner regression model. ** Genotype, age, and sex were included in the linear regression model.

Table A2 shows the cardiometabolic phenotypes that strongly associated with VDR SNP Taq1, Fok1, and Apa1 in SPS biobank adults. Genotype frequencies for Taq1, Fok1, and Apa1 were (AA: 586, AG: 339, GG:63), (GG: 275, GA: 507, AA: 208), and (CC: 344, CA: 440, AA: 200), respectively. Taq1 strongly associated with HbA1c in unadjusted (*p* = 0.01) and adjusted (*p* = 0.007) models. Fok1 had no strong associations. Apa1 strongly associated with BMI and WC. Strongest associations were seen for BMI in unadjusted (*p* = 0.005) and adjusted (*p* = 0.006) models.
